# Universal Hip Ultrasound Screening in Newborns: A 21-Month Prospective Observational Study in a Spoke Center

**DOI:** 10.3390/medsci13040311

**Published:** 2025-12-10

**Authors:** Neftj Ragusa, Nefer Roberta Gianotto, Virginia Deut, Chiara Mattivi, Francesca Compagno, Marta Cherubini Scarafoni, Silvia Dominici, Massimo Berger

**Affiliations:** Pediatrics and Neonatology Department, Ivrea Hospital, Piazza Credenza 2, ASLTO4, Piedmont, 10015 Ivrea, Italy

**Keywords:** Developmental Dysplasia of the Hip (DDH), hip dislocation, congenital, ultrasonography, neonatal screening, newborn, early diagnosis, spoke center

## Abstract

**Background:** Developmental dysplasia of the hip (DDH) encompasses a spectrum of neonatal hip abnormalities that, if not detected and treated early, may lead to long-term orthopedic sequelae. Universal ultrasound screening using Graf’s method has been proposed to improve early diagnosis, though its implementation remains heterogeneous in Italy. **Objectives:** This study aimed to describe the outcomes of a universal ultrasound screening program for DDH conducted in a first-level birth center in northern Italy, evaluating DDH incidence, risk factors, management outcomes, and program feasibility. **Methods:** A prospective observational study was conducted from February 2024 to October 2025 at the Ivrea birth center (Piedmont region, Italy). All consecutive live-born infants (*n* = 904) underwent hip ultrasound according to Graf’s method, between 0 and 11 weeks of age. Hips were classified as type I (normal), type IIa (physiologically immature), or type IIb–IV (pathological). Infants with type IIa hips were re-evaluated after 2–4 weeks; those with type IIb or worse were referred to pediatric orthopedics. **Results:** Of 1808 hips examined, 92% were Graf type I and 8% type IIa. After follow-up, 93% of type IIa hips matured spontaneously. Pathological DDH (Graf IIb or worse) was diagnosed in 8 infants (0.88%), of whom 75% were female; 50% had no identifiable risk factors. All affected infants were treated with harness before 12 weeks of age, with complete recovery and no late diagnoses. No infant required surgical treatment. **Conclusions:** Universal ultrasound screening for DDH was feasible and effective in a first-level birth center, ensuring early diagnosis and absence of late-presenting cases. These findings support universal screening as a safe and equitable approach to reduce DDH-related morbidity and align with national recommendations for standardized early detection programs.

## 1. Introduction

Developmental dysplasia of the hip (DDH) represents a spectrum of abnormalities affecting the neonatal hip, ranging from mild acetabular immaturity to complete dislocation. If not diagnosed and treated early, DDH can lead to long-term orthopedic complications such as gait disturbances, chronic pain, and early-onset hip osteoarthritis [1,2]. DDH frequency expected is 1.6% of the general population [3].

The spontaneous evolution of the less severe forms of DDH has not yet been fully defined because of the ethical difficulty of performing a randomized clinical trial involving the comparison between the “treated” and the “untreated” groups. However, retrospective observational studies available in literature report that DDH, when not detected and treated promptly, leads to an increased risk of corrective surgery and hip replacement surgery [4]. The treatment effectiveness is maximized when it begins earlier due to a not yet consolidated anatomical alterations secondary to the dislocation of the femoral head in the first 2–3 months of life of the infant [4]. Furthermore, any minimal residual alteration of the acetabulum, most likely in late treatments, may lead to hip osteoarthritis in adulthood [5]. Another notable aspect regards complications related to DDH treatment, such as avascular necrosis of the femoral head, which are more frequent in cases of late diagnosis and treatment [6,7]. Clinical examination alone, particularly in the early neonatal period, has limited sensitivity and specificity, which has prompted the increasing adoption of ultrasound screening based on standardized methods such as Graf’s [8,9]. Studies comparing the results of clinical and ultrasound examinations have shown that ultrasound screening is more sensitive to detect all children with DDH [10,11]. The concordance between clinical and ultrasound examinations is suitable in severe DDH cases (type III and IV according to Graf classification), but unsatisfactory for less severe cases (type IIb, IIc and D) [11].

While selective hip ultrasound screening for DDH based on clinical risk factors remains common in many healthcare systems, recent evidence supports the implementation of universal hip ultrasound screening for DDH [12]. A recent meta-analysis found that universal hip ultrasound screening for DDH was associated with a lower incidence of late DDH diagnoses compared to selective screening (0.10 vs. 0.45 per 1000 live births), though with an increase in abduction bracing treatment rates [13]. Another meta-analysis confirmed a similar trend, emphasizing the trade-off between early detection and overtreatment [14]. On the contrary, a 2022 systematic review and meta-analysis found that universal screening may cause initial overtreatment without reducing the rates of late detection and operative treatment [15]. Country-specific evaluations—such as in Taiwan and Austria—have demonstrated the effectiveness and feasibility of universal protocols using Graf’s method [16,17], while a UK-focused review highlighted the variability in screening practice and the need for standardization [18]. Additionally, a critical review underscored the potential cost-effectiveness of universal hip ultrasound screening for DDH in reducing long-term morbidity [19]. In Italy, neonatal screening practices for developmental dysplasia of the hip are heterogeneous and influenced by regional protocols rather than a unified national program. An Italian working group intersociety consensus (dated 2020) recommends including all newborns in a universal hip ultrasound screening for DDH program performed between 4 and 6 weeks of life, regardless of the presence of risk factors [4]. Despite these recommendations, most Italian regions still follow a selective screening model, leading to notable variability in early diagnosis and treatment approaches. However, pilot initiatives—such as the universal hip ultrasound screening for DDH model launched by the Umbria region—have demonstrated feasibility and potential cost-effectiveness, prompting advocacy for the inclusion of universal hip ultrasound screening for DDH in the National Essential Levels of Care (LEA) [20].

In our first-level birth center of Ivrea, in the Piemonte region, northern Italy, which handles approximately 600 deliveries per year, we implemented a universal hip ultrasound screening for DDH program using Graf’s method as part of routine neonatal care. The present study aimed to describe the clinical and ultrasound findings collected over a 21-month period, evaluate the incidence and severity of DDH in our population, and compare our findings with recently published data. A secondary objective was to assess the management strategies and outcomes associated with early detection in a first-level setting, contributing real-world evidence to the ongoing debate on the feasibility of universal screening programs.

## 2. Materials and Methods

We conducted a prospective observational study at our first-level birth center in Ivrea, Piemonte, northern Italy, which manages approximately 600 deliveries per year and is in a mountain area 50 km from the third-level referral pediatric hospital. The study evaluated all consecutive newborns who underwent routine ultrasound screening for developmental dysplasia of the hip (DDH) over a 21-month period, from 1 February 2024 to 31 October 2025. All live-born infants during the study period were eligible for inclusion. Neonates were enrolled regardless of clinical risk factors, findings on physical examination or gestational age. Exclusion criteria included: major congenital malformations involving the musculoskeletal system, incomplete ultrasound imaging, absence of follow-up for cases requiring clinical monitoring.

Ultrasound screening was performed according to the Graf method, using pediatric high-resolution linear transducers (L 4–15 MHz, Esaote MyLabX8, Esaote S.p.A., Genova, Italy). Each examination was conducted by a pediatrician with certified training in pediatric musculoskeletal ultrasound using standardized clinical protocols. The average duration of each ultrasound assessment was approximately 10 min per patient, varying slightly depending on the anatomical region, clinical indication and patient compliance. Throughout the examination, infants were comforted by the presence of their mother or primary caregiver, who remained close to them for the entire procedure. When available and deemed helpful, a pacifier was provided to further soothe the infant. These non-pharmacological comfort measures were sufficient to facilitate patient cooperation and ensure the acquisition of high-quality imaging data.

All newborns with breech presentation or first-grade family history for DDH underwent the ultrasound examination before being discharged from the hospital, while the others underwent hip ultrasound between 4 and 11 weeks of age as part of routine postnatal follow-up. Both hips were evaluated in the coronal plane in the standard lateral decubitus position. At least two different images per hip were systematically collected and retrospectively measured. Hips were classified according to Graf classification into type I (mature hip), type IIa (physiologically immature), type IIb-IIc-D (pathological or dysplastic), type III–IV (subluxated or dislocated). All immature hips in infants more than 3 months of age had been classified as IIb or worse, according to Graf’s criteria. Additionally, infants who demonstrated immature hips (Graf type IIa) between 10 and 12 weeks of age were defined as “type IIa at high risk of progressing to type IIb” (HR-IIa).

**Figure 1:**
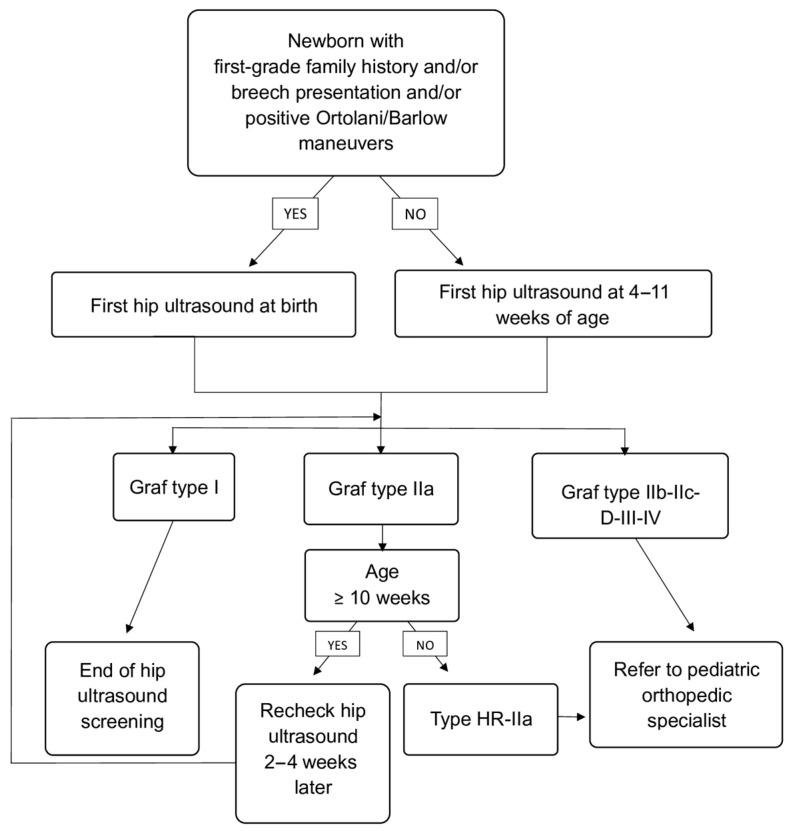
Flowchart of screening and follow-up pathway.

Infants with Graf type I hips were considered normal and discharged from further follow-up. Infants with type IIa hips were scheduled for repeat ultrasound 2–4 weeks after to assess for spontaneous maturation. In accordance with our institutional screening protocol, Graf type IIa hips were not further subdivided into IIa+ and IIa− because both undergo the same initial management, consisting of scheduled follow-up ultrasound and clinical observation. All infants with type HR-IIa, IIb or worse were referred to a pediatric orthopedic specialist for further evaluation and management, including possible initiation of abduction therapy (e.g., Pavlik harness). Screening and follow-up algorithm is shown in Figure 1.

In cases where the two hips belonging to the same infant received different Graf classifications, the follow-up pathway was determined by the hip with the more severe (i.e., more immature or pathological) classification. This conservative approach ensured that infants with unilateral abnormalities or asymmetric maturation were managed according to the highest level of clinical caution.

All ultrasound examinations were performed by licensed pediatricians (NR, NRG and VD). All ultrasound scans were digitally stored in an external digital memory. Examinations classified as abnormal or suspicious at the initial assessment were routinely reviewed by a second experienced sonographer to ensure consistency of interpretation. In case of need to repeat the ultrasound, the examination was requested by another operator other than the first. All hips classified as pathological at our institution were subsequently reassessed at the regional referral center, where a pediatric orthopedic specialist performed a confirmatory ultrasound examination. Although no formal statistical evaluation of intra- or inter-observer variability was conducted, this two-step verification process provided a consistent qualitative assessment of agreement among readers. In cases of discrepancy between the two local readers, a conservative approach was adopted: the more severe (“worst-case”) classification was recorded to minimize the risk of false-negative results. Final diagnostic confirmation and management decisions were always made in collaboration with the orthopedic unit of the third level of Regina Margherita Children’s Hospital in Turin, Italy, in accordance with national and international recommendations [4].

Demographic and clinical data, including sex, birth weight, gestational age, mode of delivery, presentation at birth, known risk factors, and Ortolani or Barlow maneuvers at birth, were recorded. In preterm infants, hip ultrasound screening for DDH was performed at the same chronological age as in term newborns, without correction for gestational age, in accordance with local clinical practice and to ensure uniformity in the screening protocol. Risk factors included breech presentation, family history, born large for gestational age (LGA), twin pregnancy, oligohydramnios, and other lower-limb anomalies. Ultrasound findings were collected in a structured digital database. The following outcomes were analyzed: distribution of Graf’s types, prevalence of DDH (Graf IIb or worse), proportion of infants requiring follow-up or treatment, spontaneous resolution rate of type IIa hips, age at diagnosis and treatment initiation. Statistical analysis was performed using SPSS version 29.0 and Excel version 2019 software. Descriptive statistics (mean, median, standard deviation, frequency, and percentage) were used. Chi-square or Fisher’s exact tests were applied to assess associations between risk factors and DDH occurrence. A *p*-value < 0.05 was considered statistically significant. The study was conducted in accordance with the principles of the Declaration of Helsinki. According to national guidelines and institutional policy, observational studies based on anonymized data collected during routine clinical care do not require formal approval from the Ethics Committee. Therefore, no formal waiver was requested. Parents/legal guardians had previously provided the standard institutional consent for clinical care and the management of clinical data. Because this generic consent does not explicitly include research use, specific verbal informed consent for the secondary use of anonymized data for this study was obtained at the time of the hip ultrasound examination. No identifiable information was collected or used. All authors declared no conflicts of interest.

## 3. Results

Between 1 February 2024 to 31 October 2025, a total of 904 infants completed universal ultrasound screening for DDH. Overall screening adherence was 96.3%, with 3.7% of eligible infants (accounting for 35 infants) not undergoing any hip ultrasound. Among those who underwent at least one hip ultrasound evaluation, 6 patients did not complete ultrasound imaging and were lost to follow-up if required clinical monitoring, so they were excluded from our study cohort.

**Figure 2:**
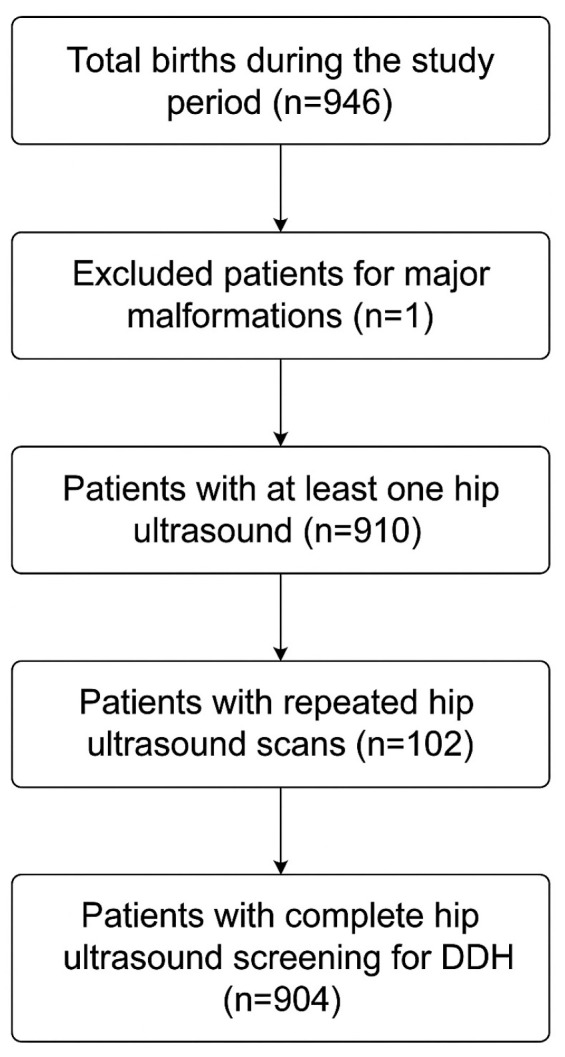
Flowchart of patients’ selection process.

The patient’s selection process is represented in Figure 2.

At the first examination, in total of 1808 hip sonograms. were evaluated using Graf’s method. The mean age at first ultrasound examination was 6.39 ± 2.23 weeks (range 0–11). Males were 476 (53%), while females were 428 (47%). In total, 38 infants (4.2%) were born preterm, with a reported gestational age ranging from 34 to 36 weeks. Among them, the first ultrasound examination was most performed at 7 weeks of chronological age.

At least one recognized risk factor for DDH was present in 171 infants (19%), 9 of whom presented two risk factors simultaneously (1%): breech presentation was reported in 34 patients (4%), positive family history in 61 patients (7%), oligohydramnios in 10 patients (1%), LGA in 66 patients (7%), twin pregnancy in 10 patients (1%), and other lower limbs anomalies in 6 patients (0.7%). On clinical examinations at birth, Ortolani or Barlow maneuvers were referred to as positive in 11 cases (1% of total cases). Fifty-two patients (6% of all) were first evaluated at birth because they presented at least one of the major risk factors for DDH, including breech presentation and first-grade family history for DDH, and/or reported positive Ortolani or Barlow maneuvers.

During the first examination, 1655 hips (92% of total), belonging to 800 infants (88.5% of all patients), were classified as Graf type I. Additionally, 149 hips (8% of all examined hips, belonging to 102 infants −11% of all patients) were identified as type IIa, 1 hip (0.06% of all scanned hip, belonging to one patient) was described as type HR-IIa, 2 hips (0.1% of all scanned hips, belonging to one patient) were described as type IIc, while none was described as type IIb, D, III or IV.

**Figure 3:**
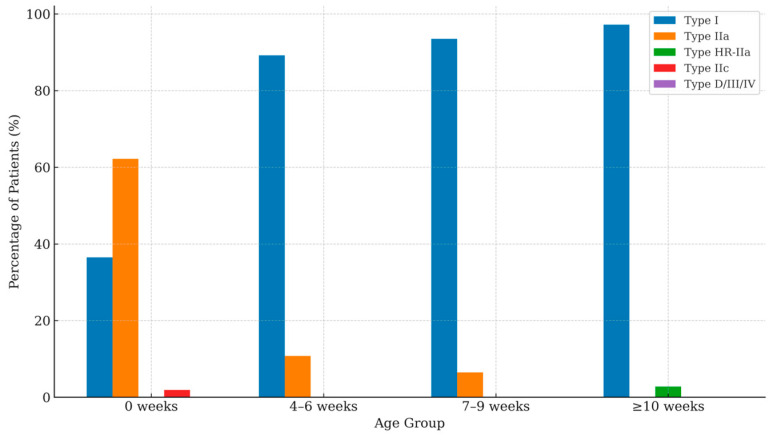
Worst-hip Graf classification at first evaluation by age group.

A stratified analysis of Graf types by age at first scan demonstrated a progressive improvement in hip morphology across age groups. At birth (0 weeks), type I hips were observed in 36.5% of infants, while type IIa represented the majority (62.2%), and type IIc accounted for 1.9%. In the 4–6-weeks group, the proportion of type I hips increased to 89.2%, with a corresponding decline in type IIa hips to 10.8%. A similar pattern was seen at 7–9 weeks, where 93.5% of infants exhibited type I hips and 6.5% remained type IIa. Beyond 10 weeks of age, 97.2% of infants demonstrated type I morphology, and 2.8% were classified as type HR-IIa. Overall, no type IIc hips were identified beyond the neonatal period. Table 1 and Figure 3 resume these results.

By the second evaluation performed 2–4 weeks after the first one, 127 type IIa hips (85% of all initial type IIa hips, belonging to 84 infants) had fully matured into type I, while 15 of them (10% of all initial type IIa hips, belonging to 13 infants) were still classified as IIa requiring a third hip ultrasound, and 7 hips (5% of all initial type IIa hips, belonging to 5 patients) were classified as HR-IIa or worst requiring orthopedic referral. By the third evaluation, 12 type IIa hips (12% of all initial type IIa hips, belonging to 10 patients) matured into type I, while 3 of them (belonging to 3 patients) were classified as HR-IIa or worst and referred to pediatric orthopedic. Table 2 shows hips distribution according to Graf’s classification.

**Figure 4:**
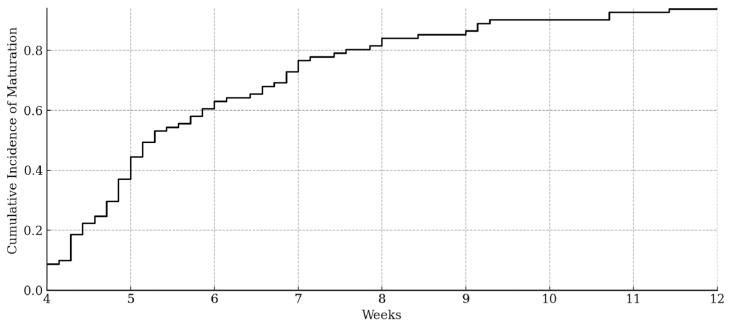
Cumulative incidence of IIa hips maturing to type I over time.

Figure 4 represents the cumulative incidence of type IIa hips maturing to type I over the first 12 weeks. The curve shows a progressive and continuous increase over the observation period. From 4 weeks, the incidence rose steadily. The curve exhibits a marked ascent between weeks 4 and 7, during which most maturation events occurred. Beyond week 9, the slope gradually flattened, indicating fewer new maturations.

Among the 10 infants (1% of all patients who completed hip ultrasound screening for DDH) referred to a pediatric orthopedic specialist, 8 (0.9% of all initially evaluated patients) were confirmed with a diagnosis of DDH and treated with a harness within 12 weeks of age, and none of them was subsequently lost to follow-up. Among confirmed DDH cases, females were 6 (75%), and males were 2 (25%). Four of them reported a bilateral DDH, while four cases presented a unilateral form. Three confirmed DDH patients reported one risk factor (two born LGA and one with positive family history), one of them reported two risk factors (breech presentation and positive family history), and four of them (50%) reported no risk factors on personal medical history. The Ortolani maneuver was negative in all cases, while the Barlow maneuver was described as positive in only one case. Table 3 presents the patient characteristics for both the DDH and non-DDH groups.

In our cohort, all the hips demonstrated satisfactory development after treatment with harness, and none required a hip spica or surgical osteotomy, as shown in Table 4. Notably, no cases of DDH were diagnosed after 12 weeks of age, with no late diagnoses within the follow-up period. This was possible thanks to collaboration with orthopedic colleagues and family pediatricians.

The overall cost of implementing the universal ultrasound screening for DDH program during the 21-month observational period amounted to €33,910.

## 4. Discussion

This prospective observational study evaluated the implementation of a universal ultrasound screening program for developmental dysplasia of the hip (DDH) in a first-level birth center. Over a 21-month period, more than 900 neonates were examined with Graf’s method, yielding a comprehensive picture of hip maturity in this population. The incidence of pathological DDH in our cohort (0.88%) was consistent with the rates reported in large population-based studies of universal screening, which typically range between 0.5% and 1.5% for Graf type IIb or worse hips [8,9,12,21]. The proportion of immature hips (physiologically immature Graf type IIa) was 11%, in line with reports from the literature, where values between 8% and 12% are typically described in universal screening populations [6,9]. As in prior studies, most of these immature hips (*n* = 139, 93%) showed spontaneous resolution at follow-up within 12 weeks of age, confirming the benign natural history of type IIa findings when monitored appropriately. Regarding risk factors, international studies consistently show that breech presentation, family history, and female sex are significantly associated with pathological DDH [2,15]. In our dataset, no significant association was detected, probably due to the very low prevalence of pathological cases (*n* = 8). Nevertheless, we found a predominance of female infants among pathological cases (75%). The higher susceptibility of females to DDH is well documented and is commonly attributed to hormonal and mechanical factors [2,15]. In our series, traditional risk factors such as Breech presentation and family history were present in half of the affected infants, while the other half had no identifiable risk factors. This finding underscores a major limitation of selective screening strategies, which would have failed to detect 50% of the DDH cases in our population. Another relevant aspect of this study is the demonstration that universal screening is feasible even in a first-level, low-volume birth center. The examinations were performed by pediatricians with specific ultrasound training, highlighting the importance of adequate operator expertise and standardization according to Graf’s technique. The collaboration with a tertiary pediatric orthopedic unit ensured appropriate management of referred cases and may serve as a model for regional network organization. The absence of severe cases (Graf type III–IV) at diagnosis further supports the benefit of early systematic screening in preventing progression to advanced forms. From a clinical standpoint, the most relevant observations were the absence of late diagnoses (later than 12 weeks of age) and the absence of hips requiring surgery in our population, supporting the effectiveness of a universal screening strategy in preventing missed cases. This aligns with Austrian and Taiwanese experiences, where universal screening protocols have successfully reduced the rate of late-presenting DDH without substantially increasing overtreatment [16,17]. The quality of our screening was then confirmed by family physicians, where no cases of DDH were later diagnosed within the follow-up period. From a public health perspective, although universal hip ultrasound screening for DDH may initially increase the number of ultrasound examinations, it may lead to the potential reduction in late diagnoses, surgical procedures, and lifelong disability, compensating for the upfront costs. This aligns with prior cost-analysis studies suggesting that universal screening represents a sustainable and effective preventive strategy [19].

The main limitation of this study is the relatively small sample size and the low number of pathological DDH cases, which limited the power of statistical analyses regarding risk factors. In addition, this is a single-center study, and our results may not be directly generalizable to populations with different demographic or healthcare characteristics. Finally, the follow-up period was limited to the first months of life, and long-term outcomes after conservative treatment were not assessed. We recognize that the relatively short follow-up period may limit the identification of late-presenting cases. All cases were analyzed, and the possibility of false positives in the Graf type I population is very remote, as also documented by larger case studies. In addition, we acknowledge that the false-negative rate of universal ultrasound screening for DDH is not yet fully established and should be further investigated in studies with extended follow-up. On the other hand, the strength of our study is given by the closer collaboration with the referring hospital and with family physicians.

Despite these limitations, our results add to the growing field of real-world evidence suggesting that universal ultrasound screening can be feasibly and effectively implemented in smaller birth centers, ensuring early detection of borderline cases and minimizing the risk of late DDH diagnosis.

## 5. Conclusions

In conclusion, the implementation of a universal hip ultrasound screening for the DDH program in a first-level birth center proved to be feasible. No late diagnoses or surgical cases were observed in our small cohort. These findings are consistent with potential benefits of early universal screening, supporting the adoption of universal screening program as recommended by national guidelines and emphasizing its potential to reduce DDH-related morbidity and promote equitable neonatal care across regional healthcare settings.

## Figures and Tables

**Table 1:** Age-stratified distribution of worst hip Graf type per patient at first evaluation.

Age Group	Type I	Type IIa	Type HR-IIa	Type IIc	Type D/III/IV
0 weeks (*n*, % of patients)	19 (36.5)	33 (62.2)	0 (0)	1 (1.9)	0 (0)
4–6 weeks (*n*, % of patients)	341 (89.2)	41 (10.8)	0 (0)	0 (0)	0 (0)
7–9 weeks (*n*, % of patients)	405 (93.5)	28 (6.5)	0 (0)	0 (0)	0 (0)
≥10 weeks (*n*, % of patients)	35 (97.2)	0 (0)	1 (2.8)	0 (0)	0 (0)

**Table 2:** Hips distribution is according to Graf’s classification.

Graf’s Classification	After First Evaluation	After Second Evaluation	After Third Evaluation
I (*n*, % of hips)	1655 (92)	127 (7)	12 (1)
IIa (*n*, % of hips)	149 (8)	15 (1)	0 (0)
HR-IIa (*n*, % of hips)	1 (0.06)	7 (0.5)	3 (0.1)
IIb (*n*, % of hips)	0 (0)	0 (0)	0 (0)
IIc (*n*, % of hips)	2 (0.1)	0 (0)	0 (0)
IId (*n*, % of hips)	0 (0)	0 (0)	0 (0)
III (*n*, % of hips)	0 (0)	0 (0)	0 (0)
IV (*n*, % of hips)	0 (0)	0 (0)	0 (0)

**Table 3:** Patients’ characteristics of the Non-DDH and DDH groups.

	Non-DDH Patients	DDH Patients	*p*-Value ^a^
Number of patients	896	8	–
Number of hips	1796	12	–
Bilateral DDH patients	–	4	–
Female (*n*, % of patients)	422 (47)	6 (75)	0.39 ^b^
Positive family history (*n*, % of patients)	59 (7)	2 (25)	0.13 ^b^
Breech presentation (*n*, % of patients)	33 (4)	1 (12)	0.34 ^b^
LGA or macrosomia (*n*, % of patients)	65 (7)	2 (25)	0.13 ^b^
Oligohydramnios (*n*, % of patients)	10 (1)	0 (0)	–
Twin pregnancy (*n*, % of patients)	10 (1)	0 (0)	–
Other lower limb malformations (*n*, % of patients)	6 (0.7)	0 (0)	–
Positive Ortolani maneuver (*n*, % of patients)	11 (1)	0 (0)	–
Positive Barlow maneuver (*n*, % of patients)	0 (0)	1 (12)	–

^a^ Statistics were performed per patient; ^b^ Fisher’s exact test; DDH: Developmental dysplasia of the hips.

**Table 4:** Outcome of orthopedic treatment in confirmed DDH cases.

Treatment	*N* (% of DDH Patients)
Pavlik harness	8 (100)
Hip spica	0 (0)
Surgical osteotomy	0 (0)

## Data Availability

The original contributions presented in this study are included in the article. Further inquiries can be directed to the corresponding author.
